# Ibrutinib Prevents Acute Lung Injury via Multi-Targeting BTK, FLT3 and EGFR in Mice

**DOI:** 10.3390/ijms232113478

**Published:** 2022-11-03

**Authors:** Huanan Rao, Xiaominting Song, Jieting Lei, Peng Lu, Guiying Zhao, Xin Kang, Duanna Zhang, Tingrui Zhang, Yali Ren, Cheng Peng, Yuzhi Li, Jin Pei, Zhixing Cao

**Affiliations:** 1 State Key Laboratory of Southwestern Chinese Medicine Resources, School of Pharmacy, Chengdu University of Traditional Chinese Medicine, Chengdu 611137, China; 2 Basic Medical College, Chengdu University of Traditional Chinese Medicine, Chengdu 611137, China

**Keywords:** ALI, ibrutinib, BTK, FLT3, EGFR, poly I:C, LPS

## Abstract

Ibrutinib has potential therapeutic or protective effects against viral- and bacterial-induced acute lung injury (ALI), likely by modulating the Bruton tyrosine kinase (BTK) signaling pathway. However, ibrutinib has multi-target effects. Moreover, immunity and inflammation targets in ALI treatment are poorly defined. We investigated whether the BTK-, FLT3-, and EGFR-related signaling pathways mediated the protective effects of ibrutinib on ALI. The intratracheal administration of poly I:C or LPS after ibrutinib administration in mice was performed by gavage. The pathological conditions of the lungs were assessed by micro-CT and HE staining. The levels of neutrophils, lymphocytes, and related inflammatory factors in the lungs were evaluated by ELISA, flow cytometry, immunohistochemistry, and immunofluorescence. Finally, the expression of proteins associated with the BTK-, FLT3-, and EGFR-related signaling pathways were evaluated by Western blotting. Ibrutinib (10 mg/kg) protected against poly I:C-induced (5 mg/kg) and LPS-induced (5 mg/kg) lung inflammation. The wet/dry weight ratio (W/D) and total proteins in the bronchoalveolar lavage fluid (BALF) were markedly reduced after ibrutinib (10 mg/kg) treatment, relative to the poly I:C- and LPS-treated groups. The levels of ALI indicators (NFκB, IL-1β, IL-6, TNF-α, IFN-γ, neutrophils, and lymphocytes) were significantly reduced after treatment. Accordingly, ibrutinib inhibited the poly I:C- and LPS-induced BTK-, FLT3-, and EGFR-related pathway activations. Ibrutinib inhibited poly I:C- and LPS-induced acute lung injury, and this may be due to its ability to suppress the BTK-, FLT3-, and EGFR-related signaling pathways. Therefore, ibrutinib is a potential protective agent for regulating immunity and inflammation in poly I:C- and LPS-induced ALI.

## 1. Introduction

Acute lung injury (ALI) is a clinical syndrome that is caused by various pathogenic factors, including infectious agents (viruses, bacteria, and plasmodium), chemical agents (oleic acid, bleomycin, paraquat, CO, phosgene, and PM2.5), and physical effects (trauma) [1,2,3,4,5,6]. ALI poses a serious threat to human health, and its treatment options involve fluid management, mechanical ventilation, anti-inflammatory medication, and antioxidant therapy. Currently, there are no effective treatment options for ALI [7,8,9].

Viral- and bacterial-associated lung infections are a major threat to global health. Influenza A viruses (H1N1, H5N1, and H7N9) and coronaviruses (SARS-CoV, SARS-CoV-2, and MERS-COV) are highly pathogenic to humans and have caused serious global pandemics. The ongoing COVID-19 global pandemic is caused by SARS-CoV-2 infection [10,11,12,13,14], which initially leads to acute lung injury (ALI) and, in some patients, the rapid progression to acute respiratory distress syndrome (ARDS) and or death [15,16,17]. Poly-riboinosinic-polyribocytidilic acid (poly I:C), a synthetic analog of dsRNA, is produced by many viruses during their replicative cycles [18,19,20]. Treating model animals with poly I:C induces ALI, which is characterized by the release of neutrophils, pulmonary inflammatory cell infiltration, myeloperoxide (MPO) diffusion out of cells, and TNF-α secretion [21,22].

Severe sepsis and septic shock are lethal complications of bacterial infections [23,24] that can cause ALI and ARDS. The most common pathogenic bacteria are Gram-positive bacteria (*Staphylococcus aureus*) and Gram-negative bacteria (*Escherichia coli* and *Pseudomonas aeruginosa*). Lipopolysaccharides (LPS) from Gram-negative bacteria are widely used to model ALI because they induce alveolar macrophage necrosis and pro-IL-1α secretion, which activate EC via IL-1/MyD88 signaling, leading to neutrophil recruitment and vascular leakage [25,26]. Biologically, LPS induces NFκB activation [27,28,29]. LPS-induced ALI involves several pathological processes, including pneumonedema, neutrophil recruitment, vascular leakage, ACE2 downregulation, and cytokine storms.

Bruton tyrosine kinase (BTK), a member of the Tec kinase family, is a critical mediator of B-cell antigen receptor (BCR)-signaling, as well as activation, and is expressed by all hematopoietic cells, apart from T lymphocytes and plasma cells [30,31,32,33]. The inhibition of BTK in tissue resident macrophages significantly decreases chemokine secretion by suppressing the LPS-induced activation of the AKT, NFκB and ERK signaling pathways [34,35,36,37]. A deficiency of BTK in alveolar neutrophils was shown to protect mice from lung inflammation [38,39], and BTK was also found to be markedly elevated during NK cell maturation and activation. Mechanistically, BTK promotes poly I:C-induced NK cell activation by enhancing IFN-γ secretion [40,41].

FMS-like tyrosine kinase 3 (FLT3) is a receptor-tyrosine kinase (RTK) that affects the proliferation, survival, and differentiation of hematopoietic progenitor cells [42]. FLT3 inhibition decreases the accumulation and maturation of lung cDCs to alleviate LPS-induced ALI [43]. Inhibiting FLT3 phosphorylation suppresses the expression of its downstream factors, ERK and AKT [44,45,46]. EGFR is a transmembrane receptor for ERBB and is a modulator of cell differentiation, proliferation, and survival. The inhibition of EGFR and NFκB signaling protects from LPS-induced ALI by significantly suppressing inflammatory responses and weakening alveolar, as well as vascular, permeability [47,48,49].

Ibrutinib, a first-generation BTK inhibitor that effectively inhibits B cell receptor and NFκB signal transduction [35,50], which may be beneficial to COVID-19 patients in pulmonary distress [51]. Moreover, ibrutinib inhibits FLT3 and EGFR [52,53,54,55,56]. However, the protective effects of ibrutinib against double-stranded RNA analogue-induced (poly I:C) and bacterial product-induced (LPS) ALI in mice have not been reported. In addition, the potential roles of BTK, FLT3, and EGFR kinases in protecting against ALI with ibrutinib are unclear. We investigated whether ibrutinib protects against poly I:C- and LPS-induced ALI and established the significance of the BTK-, FLT3-, and EGFR-related signaling pathways in protecting against ALI.

## 2. Results

### 2.1. Ibrutinib Ameliorated Poly I:C and LPS-Induced Acute Lung Inflammation and Pulmonary Edema

**Figure 1:**
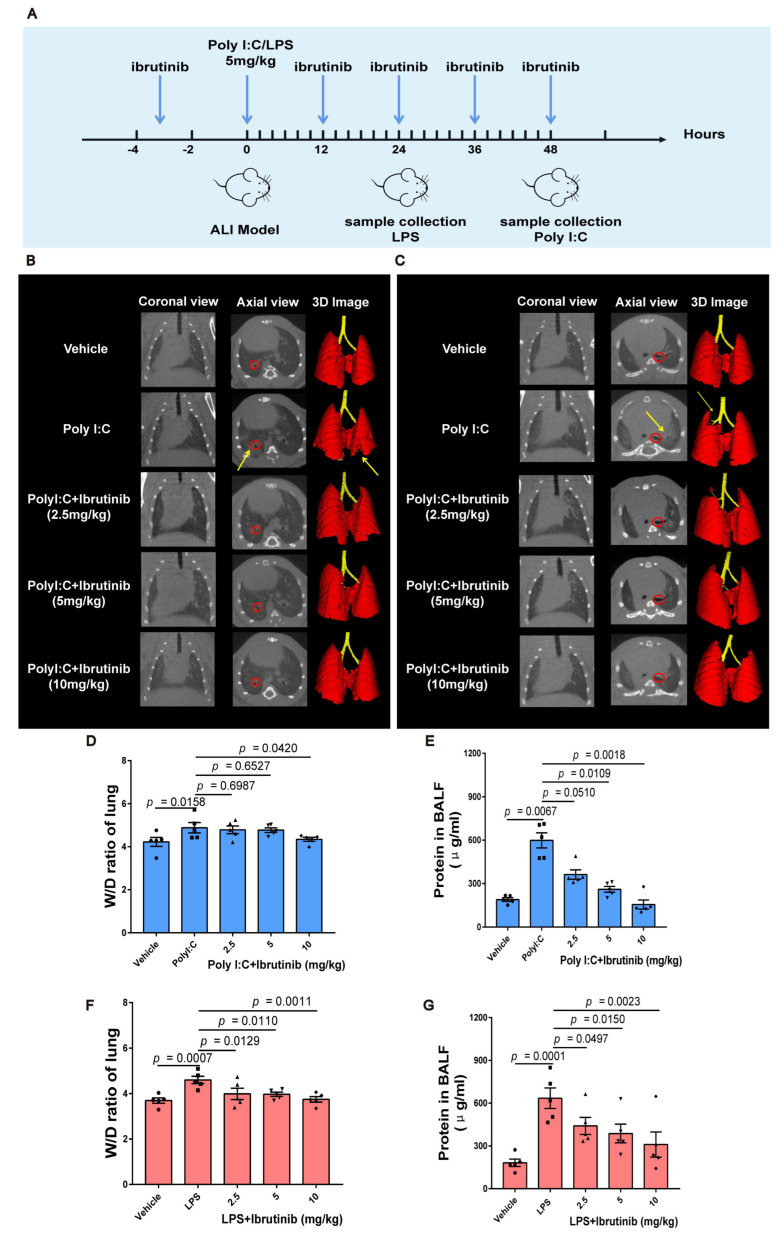
Ibrutinib protected against poly I:C- and LPS-induced acute lung injury in mice. (**A**) Schematic presentation of the experimental procedures. (**B**) Representative coronal, axial micro-CT views, and 3D lung reconstructions of the mice challenged with the vehicle, poly I:C and poly I:C plus ibrutinib (2.5, 5, and 10 mg/kg) for 48 h. (**C**) Representative coronal, axial micro-CT views, and 3D reconstructions of the lungs of mice challenged with the vehicle, LPS, and LPS plus ibrutinib (2.5, 5, and 10 mg/kg) for 24 h. (**D**) Lung tissue W/D ratios after 48 h of poly I:C challenge. (**E**) Total protein concentrations in the BALF from the poly I:C-challenged groups. (**F**) Lung tissue W/D ratios after 24 h of LPS challenge. (**G**) Total protein concentrations in the BALF from the LPS-challenged groups. Values are shown as means ± SEM for *n* = 5.

The micro-CT imaging system for small animals uses X-ray to complete three-dimensional tomography imaging, which is used in respiratory tract research imaging, and can obtain lung and bronchial images in normal or diseased states. In vivo, lung inflammation was evaluated using micro-CT every 4 h after modeling ALI. Lung injury was found to be most severe after 24 h of LPS treatment and after 48 h of poly I:C treatment. The bifurcation of the right basal segmental bronchi of the mice was marked in red to ensure selection of the same area in all CT images. High-resolution X-ray tomography images revealed large infiltrating shadows in the lower right lobes of the poly I:C-infected mice (Figure 1B). The mice that had been infected with LPS exhibited inflammatory exudation in the upper lobes of their left lungs (Figure 1C). Lung density in the ibrutinib-treated group was comparable to that of the untreated (vehicle) group, indicating that ibrutinib protected against lung injury. 3D remodeling revealed the degrees and locations of the lung injuries (Figure 1B,C).

The W/D ratio and total protein concentrations of the BALF are the key indicators for evaluating pulmonary edema in ALI [57,58]. Ibrutinib dose-dependently suppressed the increased W/D ration of the lungs and the total protein concentrations of the BALF upon challenge with poly I:C and LPS (Figure 1D–G), indicating that ibrutinib protects against poly I:C- and LPS-induced alveolar-capillary barrier dysfunction.

### 2.2. Ibrutinib Suppressed Poly I:C- and LPS-Induced Inflammation in Lungs

**Figure 2:**
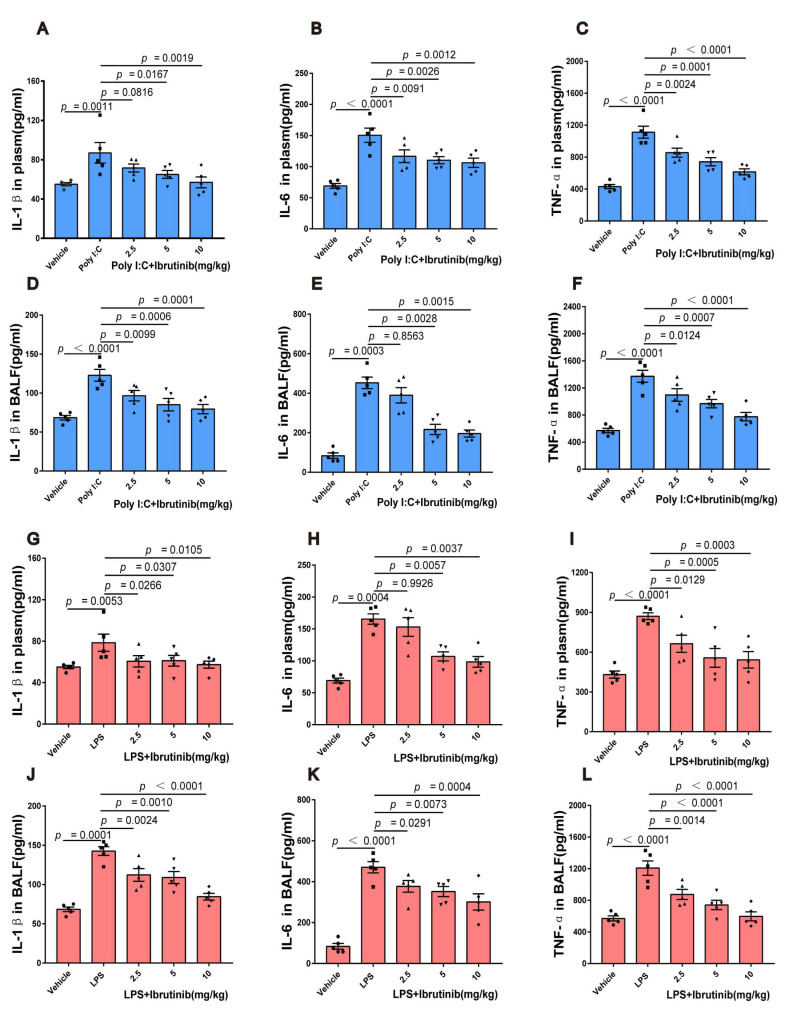
Effects of ibrutinib on inflammatory cytokine levels in poly I:C- and LPS-induced ALI. (**A**) Plasma IL-1β levels at 48 h after ALI induction. (**B**) Plasma IL-6 levels at 48 h after ALI induction. (**C**) Plasma TNF-α levels at 48 h after ALI induction. (**D**) BALF IL-1β levels at 48 h after ALI induction. (**E**) BALF IL-6 levels at 48 h after ALI induction. (**F**) BALF TNF-α levels at 48 h after ALI induction. (**G**) Plasma IL-1β levels at 24 h after ALI induction. (**H**) Plasma IL-6 levels at 24 h after ALI induction. (**I**) Plasma TNF-α levels at 24 h after ALI induction. (**J**) BALF IL-1β levels at 24 h after ALI induction. (**K**) BALF IL-6 levels at 24 h after ALI induction. (**L**) BALF TNF-α levels at 24 h after ALI induction. Values are shown as means ± SEM for *n* = 5.

Various inflammatory cytokines are responsible for poly I:C- and LPS-induced ALI, the most common and representative of which are IL-1β, IL-6, and TNF-α. Therefore, we evaluated the IL-1β, IL-6, and TNF-α levels in the plasma and BALF supernatants to assess the anti-inflammatory effects of ibrutinib during ALI. Compared to the control group, poly I:C and LPS significantly elevated the IL-1β, IL-6, and TNF-α levels in the plasma and BALF supernatant; however, ibrutinib treatment reversed this trend (Figure 2A–L). We can infer that ibrutinib inhibits poly I:C- and LPS-induced inflammatory cytokine production in the lungs.

### 2.3. Ibrutinib Inhibited Poly I:C- and LPS-Induced Neutrophil Aggregation in BALF

**Figure 3:**
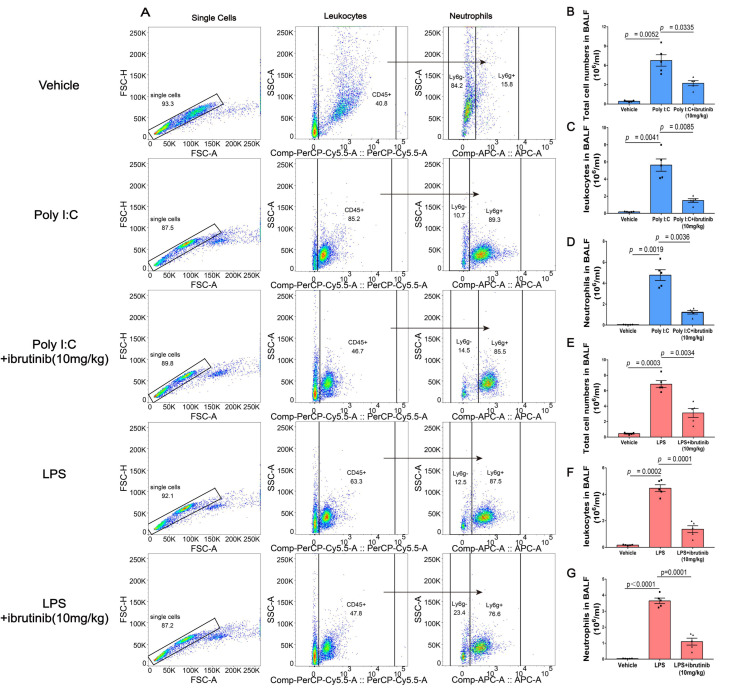
Effects of ibrutinib on poly I:C- and LPS-induced neutrophil infiltrations. (**A**) Flow cytometric plots showing the transmigratory neutrophils (Ly6G+/CD45+). (**B**) The numbers of total cells in the BALF at 48 h after ALI induction. (**C**) The numbers of leukocytes in the BALF at 48 h after ALI induction. (**D**) The numbers of neutrophils in the BALF at 48 h after ALI induction. (**E**) The numbers of total cells in the BALF at 24 h after ALI induction. (**F**) The numbers of leukocytes in the BALF at 24 h after ALI induction. (**G**) The numbers of neutrophils in the BALF at 24 h after ALI induction. Values are shown as means ± SEM for *n* = 5.

Neutrophil aggregation occurs in ALI and in its most severe type, ARDS. Poly I:C- and LPS-induced ALI heavily rely on neutrophil activation and infiltration. We assessed neutrophil infiltrations into the BALF during poly I:C- and LPS-induced ALI by evaluating the double expressions of the common leukocyte antigen (CD45) and neutrophil surface marker (Ly6G). Compared to the vehicle group, there was a significant increase in neutrophil infiltrations (CD45+/Ly6G+) in the poly I:C- and LPS-treated groups. However, neutrophil infiltrations were alleviated by 10 mg/kg ibrutinib (Figure 3A). The number of total cells, leukocytes, and neutrophils in the BALF increased after being challenged with poly I:C and LPS. However, after ibrutinib administration, this trend was reversed (Figure 3B–G)

### 2.4. Ibrutinib Attenuated Poly I:C- and LPS-Induced Lung Histopathological Changes

**Figure 4:**
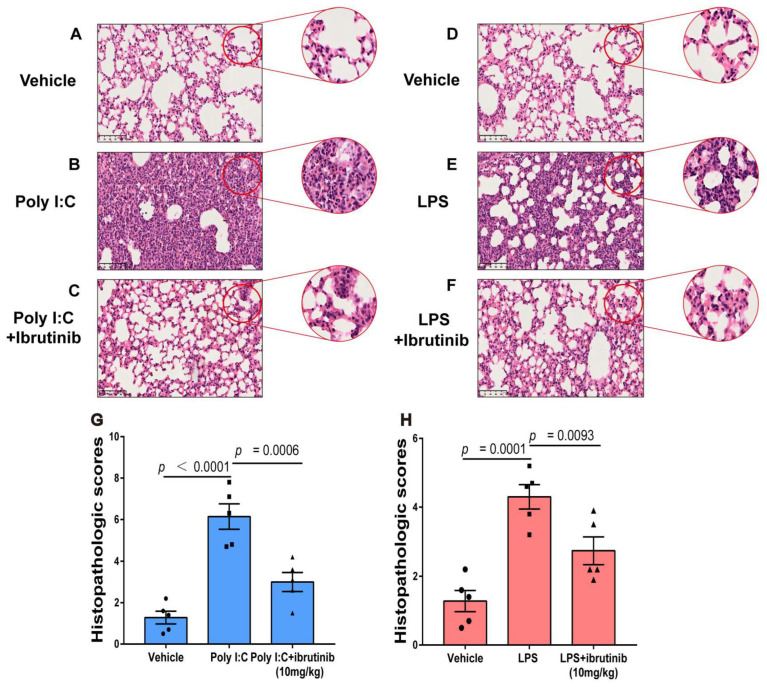
Effects of ibrutinib on poly I:C- and LPS-induced histopathological changes in lung tissues. (**A**–**C**) Representative HE images of lung sections 48 h after intratracheal poly I:C treatment. (**D**–**F**) Representative HE images of lung sections at 24 h after intratracheal LPS treatment. (**G**,**H**) Pathology scores for each group. The magnification was 200×, the scale bar = 100 μm, and all images are of the same scale. Values are shown as means ± SEM (*n* = 5).

Lung vascular endothelial cell injury has been linked to the pathogenesis of ALI. This condition is characterized by increased inflammatory cell infiltrations. In Figure 4B,E, at 48 h and 24 h of poly I:C and LPS treatments, the alveolar architectures were damaged, as revealed by interalveolar septal thickening and elevated inflammatory cell infiltrations. Although inflammation was also observed in the lung tissues of the ibrutinib-treated (10 mg/kg) mice, the level of injury was significantly lighter, inflammatory cell infiltration was lower, and lung interstitial injury was less (Figure 4C,F), indicating that ibrutinib reduces lung inflammatory reactions to poly I:C and LPS. 

Pathologically, ibrutinib significantly reduced inflammatory cell infiltrations upon being challenged with poly I:C and LPS (Figure 4G,H).

### 2.5. Ibrutinib Suppressed Neutrophil and Inflammatory Factor Levels in Poly I:C- and LPS-Induced ALI

**Figure 5:**
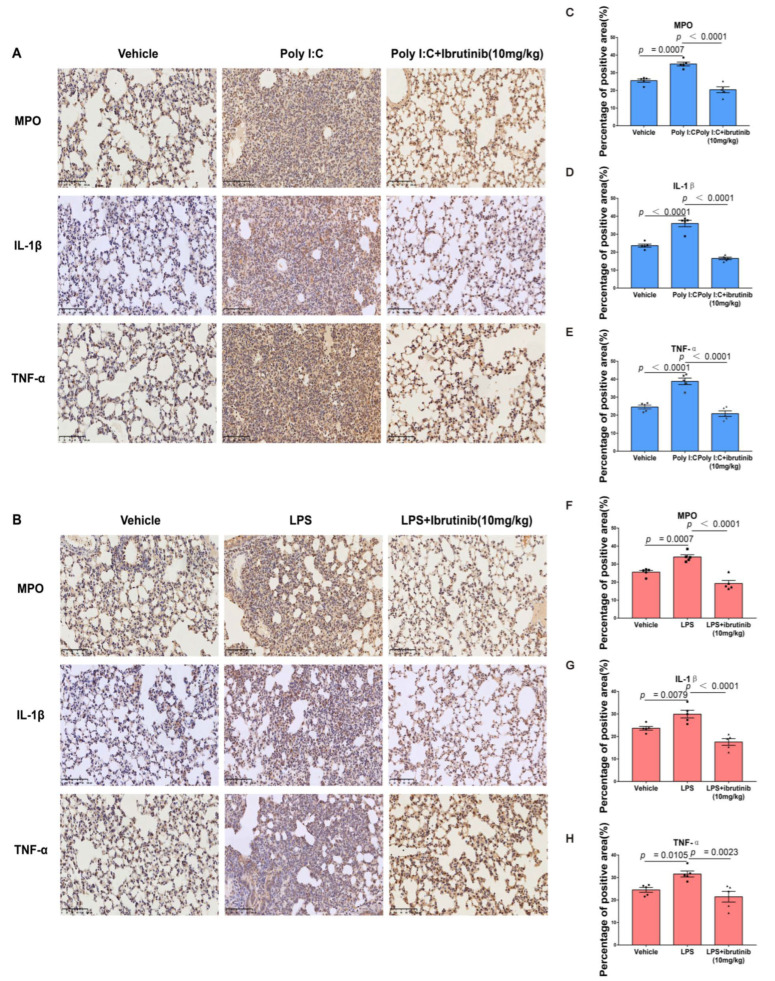
Effects of ibrutinib on the levels of MPO, IL-1β, and TNF-α in the lungs after poly I:C- and LPS-induced lung injury. (**A**) Representative immunohistochemical images of lung sections at 48 h after intratracheal treatment with poly I:C. (**B**) Representative immunohistochemical images of lung sections at 24 h after intratracheal treatment with LPS. (**C**–**H**) Proportions of MPO-, IL-1β-, and TNF-α-positive cells were semi-quantified using Image J. The magnification was 200×, the scale bar = 100 μm, and all images are of the same scale. Values are shown as means ± SEM for *n* = 5.

MPO, which is released upon neutrophil aggregation, is an indicator of neutrophil influx into inflammatory tissues [59,60]. IL-1β and TNF-α are mainly associated with pro-inflammatory immune responses [61,62,63]. To assess the protective effects of ibrutinib on poly I:C- and LPS-induced ALI, we examined the levels of neutrophil aggregation and associated inflammatory factors by measuring MPO, IL-β, and TNF-α levels. Immunohistochemical analysis revealed that MPO, IL-β, and TNF-α levels in the poly I:C and LPS groups were significantly higher relative to the vehicle control group and were mainly localized in the cytoplasm. However, ibrutinib treatment markedly suppressed their levels (Figure 5A–H), implying that ibrutinib alleviates poly I:C- and LPS-induced ALI by suppressing IL-1β, TNF-α, and neutrophil aggregation levels.

### 2.6. Neutrophil Infiltration (Ly6G) and CD8+ T-Cells Were Suppressed by Ibrutinib Treatment of Poly I:C- and LPS-Induced Lung Injury

**Figure 6:**
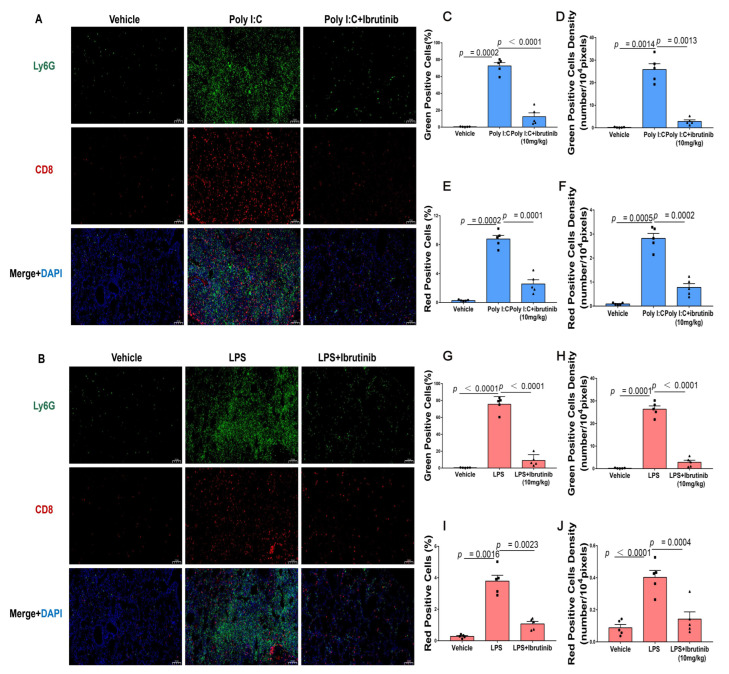
Effects of ibrutinib on poly I:C- and LPS-induced neutrophil and lymphocyte infiltrations into lungs. Ly6G (green) staining shows neutrophils, CD8 red (staining) shows T cells, and DAPI (blue) staining shows nuclei. (**A**,**B**) Representative immunofluorescence images. (**C**–**J**) Ly6G and CD8 expressions in mice lung tissues. The magnification was 200×, the scale bar = 100 μm, and all images are of the same scale. Values are shown as means ± SEM (*n* = 5).

Ly6G is a neutrophil marker that is used to label murine granulocyte MDSCs [64,65]. CD8, a cell surface glycoprotein on most cytotoxic T lymphocytes, binds class I MHC molecules and plays vital roles in immune cell–cell interactions during ALI [66,67,68,69]. We used Ly6G, a marker of murine neutrophils, to determine if poly I:C contributes to neutrophil infiltrations into lung tissues 48 h after viral infections. We found that poly I:C enhanced neutrophil infiltrations into lung tissues and that this effect was reversed by ibrutinib when compared to the negative controls (Figure 6A). Immunofluorescence analysis also revealed that 24 h after bacterial infections, ibrutinib treatment reduced neutrophil infiltrations into the lungs (Figure 6B). To characterize the physiological significance of lymphocyte infiltrations during ALI, we evaluated the CD8+ T cell levels in the lung tissues. There were marked lymphocyte infiltrations upon challenge with poly I:C and LPS, which were significantly reduced by ibrutinib treatments (Figure 6A–J). These results indicate that ibrutinib suppresses neutrophil and lymphocyte infiltrations into lungs.

### 2.7. Ibrutinib Protected from Poly I:C- and LPS-Induced ALI by Inhibiting the BTK-, FLT3-, and EGFR-Related Signaling Pathways

**Figure 7:**
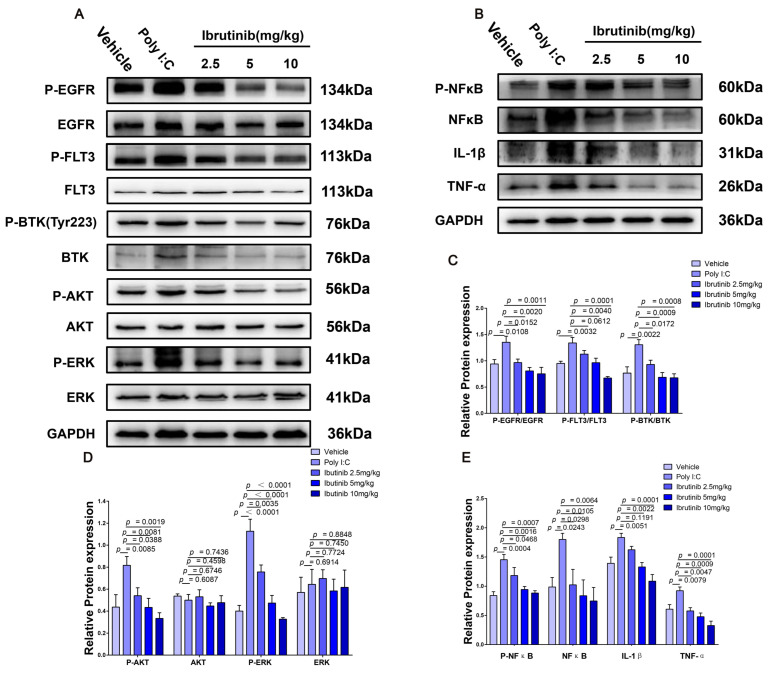
Effects of ibrutinib treatment on the BTK-, FLT3-, and EGFR-related signaling pathways during poly I:C-induced lung injury. (**A**) Protein levels of P-EGFR, EGFR, P-FLT3, FLT3, P-AKT, AKT, P-ERK, and ERK in the whole lung tissues of mice were assessed by Western blot analysis 48 h after poly I:C stimulation. (**B**) Protein levels of P-NFκB, NFκB, IL-1β, and TNF-α in the whole lung tissues of mice were assessed by Western blot analysis 48 h after poly I:C stimulation. (**C**–**E**) Densitometric analysis of the relevant bands (P-EGFR, P-FLT3, and P-BTK normalized to EGFR, FLT3, and BTK). Values are shown as means ± SEM for *n* = 3.

**Figure 8:**
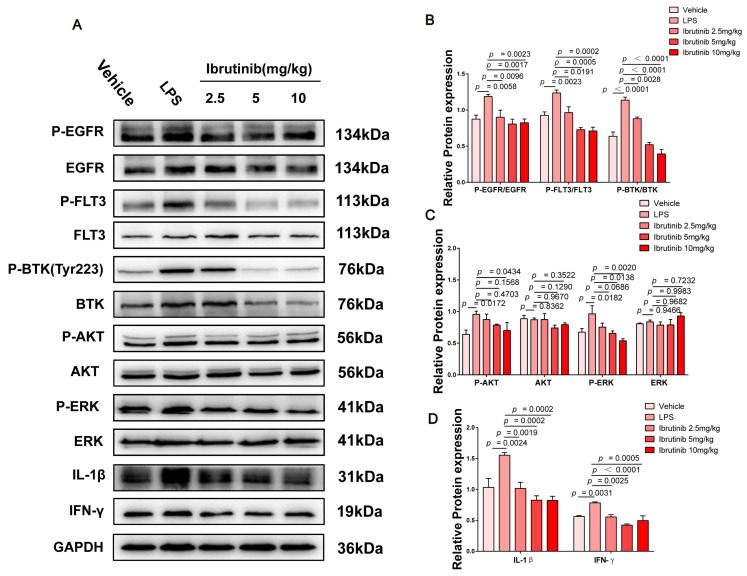
Effects of ibrutinib treatment on the BTK-, FLT3-, and EGFR-related signaling pathways during LPS-induced lung injury. (**A**) Protein levels of P-EGFR, EGFR, P-FLT3, FLT3, P-AKT, AKT, P-ERK, ERK, IL-1β, and IFN-γ in the whole lung tissues of mice were assessed by Western blot analysis 24 h after LPS stimulation. (**B**–**D**) Densitometric analysis of the relevant bands (P-EGFR, P-FLT3, and P-BTK normalized to EGFR, FLT3, and BTK). Values are shown as means ± SEM for *n* = 3.

BTK, FLT3, and EGFR play important roles during immune and inflammatory responses in mice [38,70,71,72,73]. NFκB and IFN-γ are downstream factors in the BTK-, FLT3-, and EGFR-related pathways. NFκB mediates responses to viral and bacterial infections by releasing cytokines, including IL-1β, IL-6, and TNF-α [74,75,76]. IFN-γ is produced by almost all CD8^+^ T cells, as well as by NK cells, which fight intracellular infections during ALI, and its inhibition can significantly improve viral- and bacterial-induced inflammatory responses [77,78,79,80]. To determine the mechanism by which ibrutinib protects against ALI, we quantified the expression level of proteins associated with the BTK-, FLT3-, and EGFR-related signaling pathways by Western blotting. The levels of phosphorylated EGFR, FLT3, BTK, AKT, and ERK were markedly increased after 48 h and 24 h of challenge with poly I:C and LPS, respectively. Ibrutinib dose-dependently inhibited the activation of these signaling molecules. At 5–10 mg/kg, ibrutinib significantly suppressed the BTK-, FLT3-, EGFR-, AKT-, ERK-, and NFκB-related pathways upon poly I:C challenge. The downregulation of IL-1β and TNF-α revealed that ibrutinib has antiviral inflammatory effects (Figure 7A–E). The levels of BTK, FLT3, EGFR, AKT, ERK, IL-1β, and IFN-γ were markedly increased upon LPS challenge, but they were dose-dependently suppressed by ibrutinib treatment, which significantly reduced bacterial-associated inflammation (Figure 8A–D). This indicates that the protective effects of ibrutinib on poly I:C- and LPS-induced ALI are closely associated with the BTK-, FLT3-, and EGFR-related signaling pathways.

## 3. Discussion

In this study, mice were stimulated with poly I:C and LPS to mimic viral or bacterial infections. We found that ibrutinib effectively suppressed poly I:C- and LPS-induced pulmonary edema, pro-inflammatory cytokine release, histopathological changes, neutrophil aggregation, lymphocyte aggregation, and activation of the BTK-, FLT3-, and EGFR-related signaling pathways.

BTK is a critical therapeutic target for a variety of viral and bacterial infections. Recently, it has been proved that BTK inhibitors can treat pathogen-induced ALI. Mechanistically, they signal pathways such as p38, NFκB, ERK, and AKT [81]. Ibrutinib, the world’s first listed BTK inhibitor, has been shown to have a therapeutic effect on coronavirus-induced ALI in the clinic, and it exhibited a mitigating effect on influenza-induced ALI in animal models [82,83]. In this study, we further confirmed that ibrutinib has a significant therapeutic effect on poly I:C- and LPS- induced ALI mouse models by inhibiting the abnormally activated BTK/ERK- and BTK/AKT/NFκB-signaling pathways.

Furthermore, ibrutinib is a multi-target inhibitor that could efficiently inhibit FLT3 and EGFR [84,85]. Recently, the FLT3 inhibitor (lestaurtinib) and EGFR inhibitors (erlotinib and AG1478) have been shown to protect against ALI by influencing FLT3 and EGFR kinase activities [48,86,87]. It is unknown whether ibrutinib’s efficacy against ALI is also due to its effect of targeting FLT3 and EGFR. Our experiment demonstrated that FLT3 and EGFR were hyperactivated in mouse lung tissues after stimulation with poly I:C and LPS. According to our research results, ibrutinib can effectively alleviate ALI through simultaneously inhibiting BTK, FLT3, and EGFR, as well as by suppressing the downstream signaling pathways ERK and AKT/NFκB and reducing the release of inflammatory factors such as TNF-α, IL-1β, IL-6, NFκB, and IFN-γ. Most notably, our findings indicate that FLT3 and EGFR might also be important targets for ibrutinib to protect against ALI.

LPS is a prototypical class of pathogen-associated molecular patterns (PAMPs), and it is recognized by toll-like receptor 4 (TLR4) and causes diffuse alveolar damage and neutrophil infiltration in the lungs to induce ALI [88]. It has been shown that poly I:C can act as viral PAMPs to activate toll-like receptor 3 (TLR3) to induce ALI, but poly I:C-induced ALI has rarely been reported [18]. In this study, we successfully established mice ALI models with LPS and poly I:C. We found that ibrutinib treatment improved pulmonary edema and decreased the infiltration of inflammatory cells, neutrophils, and CD8+ T cell levels in lung tissues by regulating the BTK-, FLT3-, and EGFR-related signaling pathways to alleviate ALI in mice. It is noteworthy that poly I:C could cause the hyperactivation of FLT3, and related models could be used to preliminarily evaluate the efficacy of FLT3 inhibitors in the prevention of viral lung injury.

Studies have shown that ibrutinib can affect various signaling to cause toxic and off-target adverse effects. In a previous study, ibrutinib was found to act on Csk (C-terminal Src kinase) in patients with chronic lymphocytic leukemia (CLL), thereby increasing the risk of off-target cardiotoxicity [89,90]. However, this off-target phenomenon mainly occurs in cancers such as CLL and mantle cell lymphoma (MCL), which is especially due to long-term administration. We speculate that the toxicity associated with ibrutinib is dependent on the dosage and usage cycle. Therefore, ibrutinib should be administered in low doses and for short periods of time to minimize its negative consequences.

Clinically, ALI is one of the most difficult diseases to treat. Early symptoms of severe COVID-19 are similar to those of ALI and ARDS. Several studies have investigated small molecule compounds for the treatment of COVID-19. Recently, the BTK inhibitor baricitinib was approved by the FDA for COVID-19 treatment in combination with remdesivir. In addition, five other BTK inhibitors (acalabrutinib, abivertinib, zanubrutinib, ibrutinib, and TL-895) are undergoing phase two clinical trials in the US [91,92,93,94,95]. Further, the FLT3 inhibitor gilteritinib and EGFR inhibitor abivertinib have also shown great promising potential in treating COVID-19 [70,96,97]. Clinical reports have shown that ibrutinib mainly has a potential protective effect on Waldenstrom macroglob-ulinemia (WM) patients who were suffering in the early stages of COVID-19 infection [51]. However, the protective and therapeutic effects of ibrutinib against COVID-19 are under debate. Therapeutic strategies for COVID-19 patients are complex, and so the synergistic effects of BTK, FLT3, and EGFR in ALI should also be highlighted in the development of broad-spectrum antiviral compounds that boost the innate response.

Ibrutinib can protect patients from COVID-19 infections by regulating macrophage polarization and reducing host inflammatory responses. However, the specific mechanisms are yet to be established. In this study, we found that the protective mechanism of ibrutinib against viral- and bacterial-induced ALI involves the regulation of the BTK-, FLT3-, and EGFR-related signaling pathways and the suppression of inflammatory factors. We hope that our findings provide ideas for further research on the exact molecular mechanism of ibrutinib in the prevention of COVID-19. This will lead to the formulation of better clinical treatment strategies for COVID-19 and other inflammatory diseases.

## 4. Materials and Methods

### 4.1. Chemicals and Materials

Ibrutinib was purchased from Macklin (Shanghai, China). Poly I:C was purchased from APExBIO (Houston, TX, USA). LPS was purchased from Sigma (St Louis, MO, USA). Mouse IL-1β, IL-6, and TNF-α ELISA kits were procured from Solaibao (Beijing, China). Anti-myeloperoxidase mouse mAb, anti-IL1 beta rabbit pAb, anti-TNF-α rabbit pAb, anti-CD8 mouse mAb, and anti-Ly6g rabbit pAb were obtained from Servicebio (Wuhan, China). APC Anti-Mouse Ly6G Antibody [1A8] and PerCP/Cyanine5.5 Anti-Mouse CD45 Antibody [30-F11] were purchased from Elabsciense (Shanghai, China). Antibodies against BTK/p-BTK (Tyr223), ERK/p-ERK, AKT/p-AKT, FLT3/p-FLT3, and EGFR/p-EGFR were bought from Cell Signaling Technology (Beverly, MA, USA). Antibodies against GAPDH, NFκB/p-NFκB, IL-1β, IL-6, IFN-γ, and TNF-α were purchased from Affinity Biosciences (Inc., Cincinnati, OH, USA). The RIPA lysis buffer and a Bradford protein assay kit were purchased from Biyuntian Biotechnology (Shanghai, China). PBS was purchased from Zhongshan Jinqiao Biotechnology (Co., Ltd., Beijing, China). A SuperLumia ECL HRP kit was procured from Abbkine (Redlands, CA, USA).

### 4.2. Establishment of Poly I:C and LPS-Induced ALI Model

Male BALB/c mice (20–25 g) were purchased from Chengdu Dashuo Biotechnology Co., Ltd. and housed in specific pathogen-free (SPF) conditions with access to water and chow. All experimental protocols adhered to the animal care and use guidelines of the National Institutes of Health, and they were approved by the animal care and use committee of Chengdu University of Traditional Chinese medicine (ethical code: CDU2022S1025).

A total of 135 mice were randomized into 9 groups: the vehicle group, poly I:C model group, poly I:C plus ibrutinib (2.5 mg/kg) group, poly I:C plus ibrutinib (5 mg/kg) group, poly I:C plus ibrutinib (10 mg/kg) group, LPS model group, LPS plus ibrutinib (2.5 mg/kg) group, LPS plus ibrutinib (5 mg/kg) group, and LPS plus ibrutinib (10 mg/kg) group. The mice were anesthetized via intraperitoneal injection of 100 μL/10 g of 0.4% pentobarbital, followed by the intratracheal administration of poly I:C (5 mg/kg), LPS (5 mg/kg), or a similar volume of PBS. Ibrutinib (2.5, 5, and 10 mg/kg) was administered by gavage 2–4 h prior to establishing the poly I:C or LPS models and once every 12 h after treatment with poly I:C and LPS. After 48 or 24 h, the materials needed for the experiment were obtained, respectively.

### 4.3. In Vivo Micro-CT Imaging

The mice were intraperitoneally administered 0.4% pentobarbital (100 μL/10 g) at 24 and 48 h and examined by micro-CT imaging on a Quantum GX micro-CT (PerkinElmer, Inc., Waltham, MA, USA). The scan modes were X-ray: kV:70 kV and X-ray μA: 80 μA, with a scan time of 4 min, an FOV of 36 mm, and a pixel size of 72.0 μm. The images were analyzed using Analyze 12.0 and 3D representations reconstructed.

### 4.4. Pulmonary Edema Assessment

After scanning, the right lungs were resected and washed using sterile saline, and their wet weights were determined. Then, they were dried for 48 h in an oven at 65 °C and weighed again. Edema degrees were calculated based on lung wet/dry weight ratio (W/D) using the formula: (wet weight/dry weight) × 100%.

### 4.5. Bronchoalveolar Lavage Fluid (BALF) Analysis

The mice were placed on a foam board and their tracheas were exposed using surgical scissors and curved forceps, and then they were cut. An indwelling needle was implanted and fixed using a thread. The lungs were lavaged twice using chilled sterile PBS (total volume 1.4 mL). The BALF recovery rate was >90%. Then, the BALF was centrifuged at 1500× *g* for 10 min at 4 °C and protein concentrations in the supernatant were measured using a Bradford assay (at an optical density of 595 nm).

Supernatants from the BALF were stored at −80 °C for cytokine analysis using ELISA. Then, the cell pellets were resuspended in 1.0 mL ice cold PBS (pH 7.4), after which hemocytometers were used to count all the BALF cells. The PerCP/Cyanine5.5 Anti-Mouse CD45 Antibody [30-F11] or APC Anti-Mouse Ly6G Antibody [1A8] were used to stain the BALF cells for the analysis of leukocytes or neutrophils by flow cytometry. We collected data on 10,000 cells using BD FACSVerse (BD Biosciences, CA, USA) and analyzed it using FlowJo 10.

### 4.6. Assessment of Cytokine Levels

The mice were fully anesthetized and their eyeballs were extracted to collect blood samples into EDTA-coated polypropylene tubes. The samples were centrifuged at 6000× *g* for 10 min to obtain plasma. Then, the IL-1β, IL-6, and TNF-α levels in the plasma and the BALF supernatants were determined by ELISA, following the manufacturer’s instructions. The results were measured using a microplate reader at 450 nm.

### 4.7. Pulmonary Histopathology

The left lungs of the mice were fixed in 4% PFA for >24 h, paraffin-embedded, sectioned, dewaxed, stained with an HE differentiation solution, dehydrated, and sealed with neutral gum. The HE-stained sections were scanned with Nanozoomer S60 (Hamamatsu, Hamamatsu City, Japan). The findings were semi-quantified by two independent pathologists, as previously reported [98,99].

### 4.8. Immunohistochemical and Immunofluorescence Analysis

The lung sections were incubated overnight with the indicated primary antibodies at 4 °C. Endogenous peroxidase activities were blocked using 3% hydrogen peroxide. Then, the tissues were incubated with HRP-conjugated secondary antibodies at room temperature for 50 min and imaged under a microscope. The images were analyzed using Image J software (NIH, Bethesda, MD, USA).

For the immunofluorescence assay, after blocking with 3% BSA solution for 30 min, the sections were incubated overnight with various primary antibodies at 4 °C. Subsequently, the sections were incubated with horseradish peroxidase (HRP) -labeled secondary antibody for 50 min and then washed with PBS. This was followed by incubation with FITC-TSA-conjugated secondary antibodies for 10 min at room temperature in darkness. The nuclei on paraffins were counterstained with DAPI. Finally, the sections were sealed using an anti-fluorescence quencher to quench spontaneous fluorescence and examined by fluorescence microscopy. The immunofluorescence of the sections was recorded under a UV excitation wavelength of 330–380 nm and an emission wavelength of 420 nm.

### 4.9. Western Blot Analysis

Whole lung tissues from the mice were snap-frozen and stored at −80 °C and quickly ground into fine powder in liquid nitrogen using a mortar and pestle. A pre-chilled protein lysate (0.1 mg/mL) and 1.3% PMSF were added into Eppendorf tubes and incubated on ice for 20–30 min. Then, they were centrifuged at 12,000× *g* for 15 min at 4 °C and the protein concentrations were determined using a Bradford assay. After adjusting the protein concentrations, 0.25% SDS of the final supernatant volume was added to the samples, boiled at 100 °C for 5 min, separated by 10% SDS–PAGE gel electrophoresis, and transferred onto PVDF membranes. Then, the membranes were blocked with 5% milk in TBST for 2 h and thereafter incubated overnight with primary antibodies against p-EGFR, EGFR, p-FLT3, FLT3, p-BTK, BTK, p-AKT, AKT, p-ERK, ERK, p-NFκB, NFκB, TNF-α, IL-1β, IL-6, IFN-γ, and GAPDH at 4 °C. They were washed, incubated with HRP-conjugated secondary antibodies, and signal developed using a SuperLumia ECL HRP substrate kit.

### 4.10. Statistical Analysis

The data are shown as means ± SEM. The parametric data were analyzed using one-way ANOVA followed by a Tukey–Kramer test. The non-parametric data were analyzed using a Kruskal–Wallis test, followed by a Dunn’s test. All analyses were performed using GraphPad Prism 7.0 software, and *p* ≤ 0.05 indicated significant differences. Specific *p*-values are described in the figure legends.

## 5. Conclusions

We used a novel viral- and bacterial-simulation-induced ALI model to prove the protective effects of ibrutinib on ALI, which are attributed to regulation of the BTK-, FLT3-, and EGFR-related signaling pathways, inhibition of the expression of downstream inflammatory factors (NFκB, IL-1β, IL-6, TNF-α, and IFN-γ), and improvement of neutrophil, as well as lymphocyte, aggregation. Ibrutinib is a promising protection and treatment option for ALI. Importantly, the therapeutic mechanism of ibrutinib on ALI involves multi-target effects, including BTK, FLT3, and EGFR, which merits further investigation.

## Data Availability

The original contributions presented in the study are included in the article, further inquiries can be directed to the corresponding author/s.
